# Protein STM3547 From *Salmonella typhimurium* Is a Phosphofructose Kinase B‐Type Enzyme With Ribose Kinase Activity

**DOI:** 10.1111/mmi.70084

**Published:** 2026-06-05

**Authors:** Regan D. McCormick, Aatif A. Jabbar, Jorge C. Escalante‐Semerena

**Affiliations:** ^1^ Department of Microbiology University of Georgia Athens Georgia USA

**Keywords:** carbohydrate metabolism, PfkB family of sugar kinases, ribose kinase, *Salmonella enterica*

## Abstract

The first step in the metabolic utilization of ribose is the phosphorylation of its 5′ hydroxyl group. The enteropathogenic bacterium *Salmonella enterica* subsp. *enterica* sv. Typhimurium str. LT2 (hereafter *S.* Typhimurium) has two known enzymes that can phosphorylate D‐ribose, namely ribose kinase (RbsK) and deoxyribose kinase (DeoK). Here, we report in vivo and in vitro evidence to support the conclusion that the previously uncharacterized STM3547 protein of this bacterium has ribose kinase activity, and we propose naming it RikA (Ribokinase A). Our bioinformatics analyses show end‐to‐end sequence identity/similarity among RikA, RbsK, and DeoK, and site‐directed mutagenesis of RikA showed that, like in RbsK and DeoK, an aspartate residue (position 343 in RikA) is critical for enzymatic activity. We found that RikA has *K*_*m*_ values comparable to those of RbsK and DeoK for both ribose and ATP, and that all three proteins display cooperative binding of ATP induced by ribose. Together, these data suggest that the ribose‐induced conformational changes observed in *E. coli* RbsK also occur in all three ribose kinases of *S. typhimurium* . Notably, while RikA has robust ribose kinase activity, it can only poorly phosphorylate 2‐deoxy‐D‐ribose, D‐xylose, and D‐xylulose, and it cannot phosphorylate L‐arabinose. Collectively, our data support the conclusion that the RikA protein is a member of the phosphofructose kinase B‐type (PfkB) family of sugar kinases.

## Plain Language Summary

Validation of the function of hypothetical genes is critical to advancing our understanding of cell physiology and metabolism. Here we identify the function of a hypothetical protein in a human bacterial enteropathogen.

## Graphical abstract

The metabolism of ribose starts with the phosphorylation of its 5′ hydroxyl group. *Salmonella typhimurium* has two kinases that perform this function, namely ribose rinase (RbsK) and deoxyribose kinase (DeoK). Here we report a new ribose kinase that we named RikA. The illustration was partially created in BioRender. Escalante‐Semerena (2026) https://BioRender.com/bezkrbn.

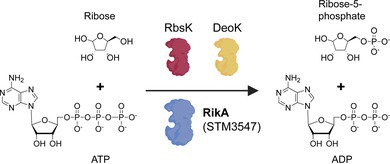

## 1 Introduction

Ribose is one of the most abundant sugars in biological systems, as it is a component of RNA, NTPs, and various other biological molecules. Before ribose can be metabolized, it must be phosphorylated. The genome of *Salmonella enterica* subsp. *enterica* sv. Typhimurium str. LT2 (hereafter, *S.* Typhimurium) encodes two proteins known to have ribose kinase activity, namely RbsK and DeoK. Although there is little research on *S.* Typhimurium RbsK, its homolog in *E. coli* has been extensively studied. The existence of a ribose kinase and its importance in ribose metabolism were first reported by Anderson and Cooper (Anderson and Cooper 1969, 1970). The *rbsK* gene was shown to be in an operon downstream of the genes encoding the ribose transporter (*rbsACB*) (Iida et al. 1984), and upstream of the transcriptional regulator (RbsR) for this operon (Lopilato et al. 1984). Crystal structures of the protein have been solved with and without its substrates in the active site (Sigrell et al. 1998, 1997, 1999; Andersson and Mowbray 2002), and RbsK kinetics and substrate specificity has been extensively studied (Maj and Gupta 2001; Chuvikovsky et al. 2006). *S.* Typhimurium has also been shown to grow on 2‐deoxy‐D‐ribose (hereafter, deoxyribose) as a sole carbon source, using a pathway that begins with the phosphorylation of D‐deoxyribose (Hoffee 1968). Purification and analysis of the deoxyribose kinase (DeoK) from *S.* Typhimurium found that it can also use D‐ribose as substrate (Schimmel et al. 1974).

**FIGURE 1:**
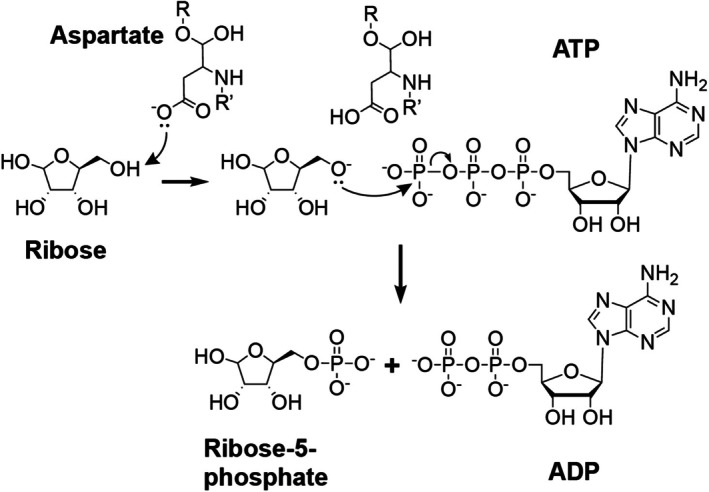
Mechanism of *E. coli* RbsK. Aspartate at position 255 of *E. coli* RbsK removes the proton from the 5′ hydroxyl group of ribose. The free electrons on the 5′ oxygen then perform a nucleophilic attack on the γ‐phosphate of ATP, and the final products are released.

*E. coli* RbsK was identified as belonging to the PfkB superfamily of carbohydrate kinases (Wu et al. 1991), and structural studies have generated insights about the reaction mechanism and substrate binding for RbsK and the family at large. Analysis of RbsK crystals in complex with D‐ribose and an ATP analog demonstrated that aspartate at position 255 acts as a base to abstract the proton from the 5′ hydroxyl group of ribose, allowing for a nucleophilic attack on the γ‐phosphate of ATP (Figure 1; Sigrell et al. 1998). Subsequent work showed that D‐ribose binding to RbsK induced conformational changes in the ATP binding pocket that may increase its affinity for ATP (Sigrell et al. 1999).

**FIGURE 2:**
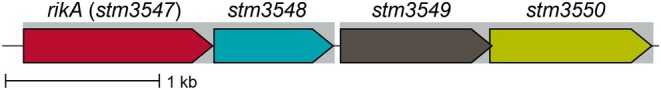
The *rikA* gene and its genetic context. The *rikA* gene (*stm3547*) is upstream of three uncharacterized genes: *stm3548*, *stm3549*, and *stm3550*. These genes are predicted to be a glutamine amidotransferase, DMT family transporter, and phosphotriesterase, respectively. Additionally, this region is predicted to be expressed as two operons (BioCyc.com, gray rectangles around gene figures).

Here, we define the ribose kinase activity of a hypothetical protein from *S.* Typhimurium encoded by the *STM3547* locus and propose to name the protein RikA. We show that the RikA protein is sufficient to support growth on D‐ribose (hereafter, ribose) as a sole carbon and energy source and provide evidence that the RikA enzyme is a member of the PfkB family of carbohydrate kinases. Moreover, we demonstrate that RikA phosphorylates ribose in vitro at the expense of ATP and describe the apparent kinetic parameters of the enzyme with ribose and ATP as substrates. The *STM3547* gene is upstream of hypothetical genes predicted to encode a glutamine amidotransferase (*STM3548*), a DMT family transporter (*STM3549*), and a phosphotriesterase (*STM3550*) (Figure 2). We also find that this genetic locus is conserved in a subset of Enterobacterales.

## 2 Results and Discussion

### 2.1 RikA Function Is Sufficient for Growth on Ribose as the Sole Carbon Source

**FIGURE 3:**
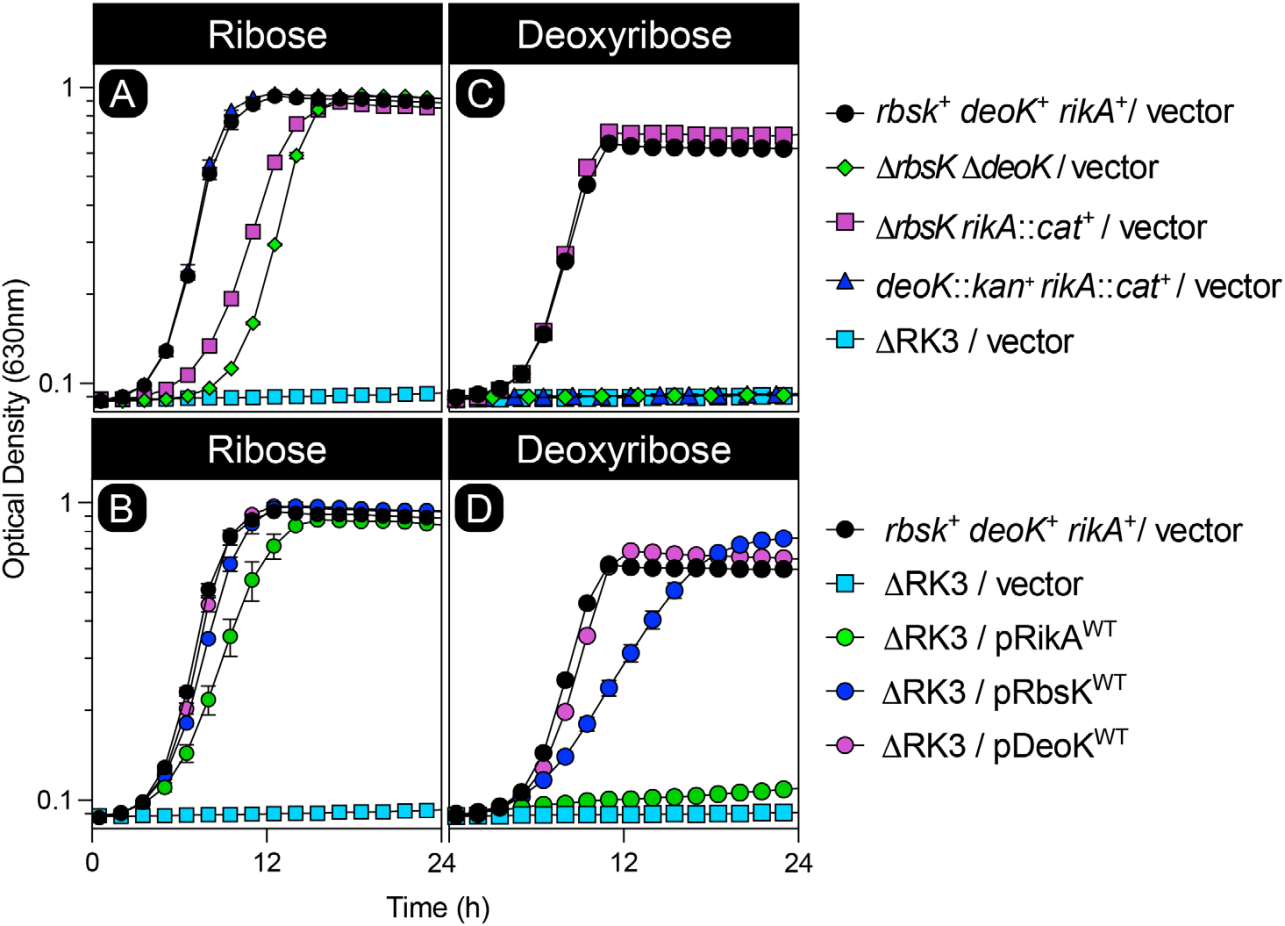
RikA function is sufficient for growth on ribose, but not deoxyribose, as the sole carbon source. Each strain was grown overnight in NB + ampicillin (100 μg/mL) at 37°C with agitation and sub‐cultured 1:100 into NCE minimal medium with either (A, B) ribose (22 mM) or (C, D) deoxyribose (22 mM) as the sole carbon and energy source. Cultures were grown in 96‐well plates at 37°C with agitation. Genetic notations are as follows: ∆*rbsK* stands for ∆*rbsK84*, *deoK::kan* ^+^ stands for *deoK407::kan* ^+^, ∆RK3 stands for the ∆*rbsK84* ∆*deoK408 rikA::cat* ^+^ triple deletion. ‘Vector’ stands for the empty cloning vector pCV1 that contains the arabinose‐inducible P_*araBAD*_ promoter (Guzman et al. 1995; VanDrisse and Escalante‐Semerena 2016), pRikA^WT^ is pRikA‐2, pRbsK^WT^ is pRbsK‐1, pDeoK^WT^ is pDeoK‐1 (see *Experimental procedures*). Plasmids were maintained with ampicillin (100 μg/mL), and ectopic gene expression was induced with L‐(+)‐arabinose (500 μM). This experiment was conducted in technical triplicate of biological duplicates. Error bars represent one standard deviation from the mean. Error bars that are not visible are smaller than the symbol.

RbsK is the primary ribose kinase in *E. coli* (Anderson and Cooper 1969), and this enzyme is presumed to have the same function in *S.* Typhimurium based on primary sequence similarity. Additionally, the deoxyribokinase enzyme from *S.* Typhimurium (i.e., DeoK) has ribose kinase activity (Schimmel et al. 1974). Bioinformatics analysis of the *S.* Typhimurium genome suggested that STM3547 (RikA) might have ribose kinase activity (Karp et al. 2019), and we created strains of *S.* Typhimurium lacking *rbsK*, *deoK*, *rikA*, and all combinations thereof. We assayed the ability of each strain to grow on ribose as a sole carbon source and found that the presence of *rbsK*, *deoK*, or *rikA* in the chromosome was sufficient for growth (Figure 3A, blue triangles, magenta squares, and green diamonds). The strain without *rbsK*, *deoK*, and *rikA* (ΔRK3) failed to grow on ribose (Figure 3A,B, cyan squares), while expressing *rikA* ^+^ from a plasmid restored growth (Figure 3B, green circles). Strains carrying chromosomal deletions of any one of the genes encoding ribose kinases grew to full density on ribose (Figure S1A), and none of the ribose kinase deletions or combinations had any defect on glucose as the sole carbon and energy source (Figure S2). Because *S.* Typhimurium RbsK has been shown to support growth of *E. coli* on deoxyribose as the sole carbon and energy source when overexpressed from a plasmid (Tourneux et al. 2000), and DeoK can support growth on ribose as a sole carbon and energy source (Figure 3A, magenta squares, Figure 3B, magenta circles), we tested whether RikA could support growth on deoxyribose. We found that RikA was not sufficient for growth on deoxyribose, either in the chromosome (Figure 3C, green diamonds), or when *rikA* ^+^ was expressed from a plasmid (Figure 3D, green circles). Moreover, our results support the previous report that RbsK from *S. typhimurium* can support growth on deoxyribose when ectopically expressed (Tourneux et al. 2000). While all strains without *deoK* in the chromosome failed to grow on deoxyribose (Figure S1B, magenta squares, Figure 3C, green diamonds, blue triangles, and cyan squares), ectopic expression of *rbsK* in the ∆RK3 strain supported growth on deoxyribose (Figure 3D, blue circles).

### 2.2 RikA Is a PfkB Family Carbohydrate Kinase

**FIGURE 4:**
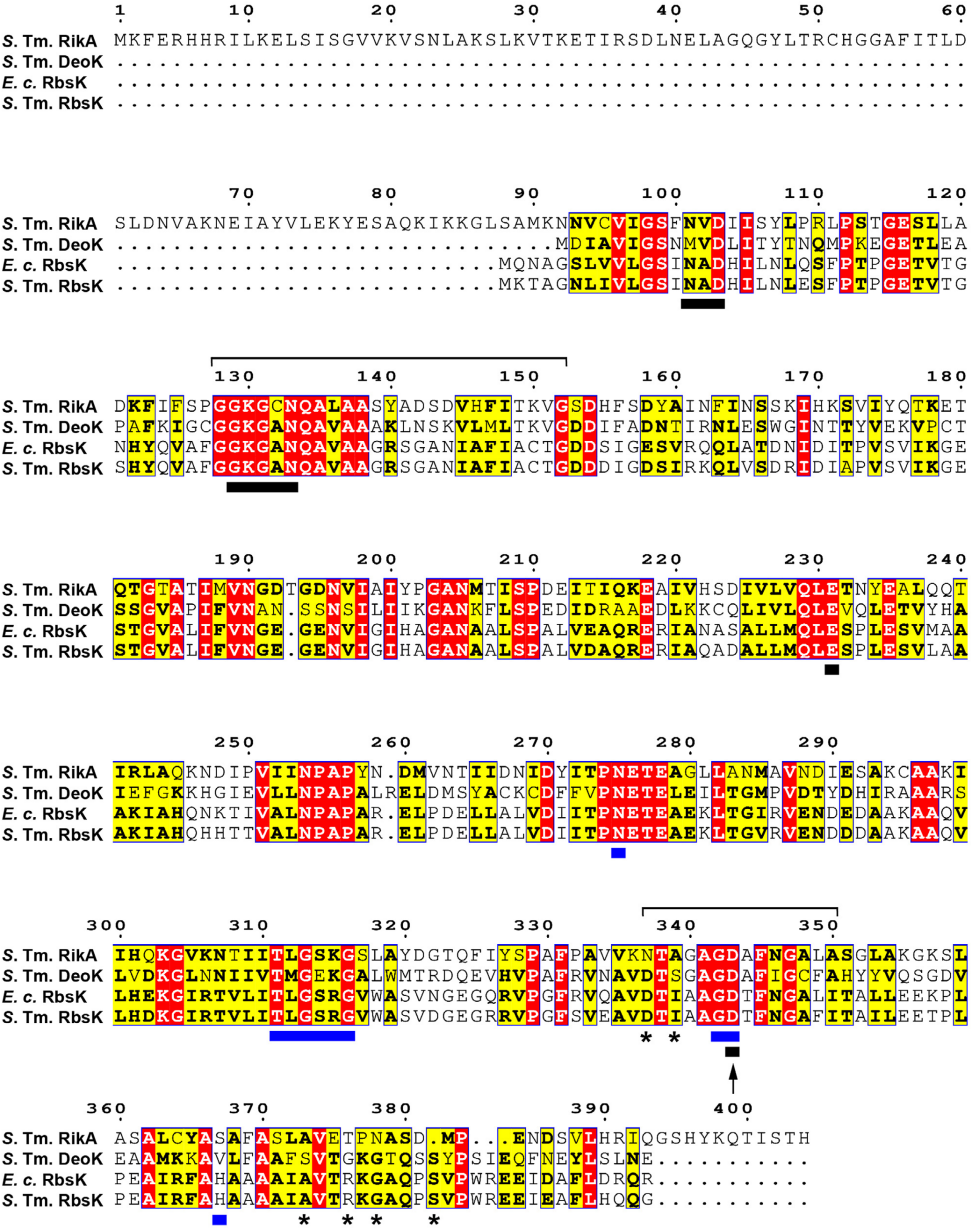
RikA is a homolog to *bona fide* members of the PfkB family of carbohydrate kinases. The amino acid sequences of *S. typhimurium* RikA, RbsK, and DeoK, and *E. coli* RbsK were obtained from BioCyc.com, aligned using Clustal Omega (Sievers and Higgins 2021), and visualized using ESPript 3.0 (flashy color setting). The shapes underneath the alignment indicate a function of those residues as follows: (i) black bars are involved in ribose binding, (ii) blue bars are involved in ATP binding, (iii) asterisks are involved potassium binding, and (iv) the arrow points at the active site aspartate. Brackets above the sequences highlight the signature patterns of PfkB family kinases (PROSITE entries P00583 and P00584). Functional annotations were taken from UniProt entry P0A9J6.

To investigate the level of conservation between the RikA, DeoK and RbsK proteins in *S.* Typhimurium we performed a multiple sequence alignment of the above proteins from *S.* Typhimurium and the RbsK protein from *E. coli* using Clustal Omega (Sievers and Higgins 2021). The results of this alignment showed a high level of conservation between the four proteins analyzed, especially in residues involved in substrate binding (Figure 4). Crystallographic studies on *E. coli* RbsK have identified 10 residues involved in binding each ribose and ATP (Figure 4, black and blue bars, respectively). Six of the ten residues implicated in ribose binding in *E. coli* RbsK are conserved in all four proteins analyzed, and eight of ten are conserved between RikA and *E. coli* RbsK. The same is true for the residues involved in ATP binding.

Moreover, the aspartate residue D255 that is required for kinase activity in *E. coli* RbsK (Sigrell et al. 1998; Andersson and Mowbray 2002) is conserved at position 343 of RikA. The active site aspartate is conserved throughout the PfkB family of carbohydrate kinases (Wu et al. 1991), and the family has two sequence regions defined as signature patterns by PROSITE. The first is a glycine‐rich sequence near the N‐terminus, and the second is near the C‐terminus. Both are involved in substrate binding, and the second contains the active site (PROSITE entries PS00583 and PS00584, respectively) (Sigrist et al. 2026). In RikA, residues 128–153 align with the PfkB family signature pattern 1 (PS00583), and residues 337–350 align with pattern 2 (PS00584). Our analysis found that RikA is 40% identical to the other three proteins in the N‐terminal signature pattern and 43% identical to the others in the C‐terminal signature pattern (Figure 4, brackets above aligned sequences). The PfkB family signature patterns, moreover, allow for variations at certain residues in the sequence. Residues 128–153 in RikA only disagree with PfkB family pattern 1 in three places, and none of the residues from RikA residues 337–350 are in conflict with pattern 2 (Figure 4, brackets vs. PROSITE entries PS00583 and PS00584). In all, sequence similarity between RikA from *S.* Typhimurium and known PfkB family carbohydrate kinases, along with the PfkB family signature patterns, indicate that RikA belongs to the PfkB family.

**FIGURE 5:**
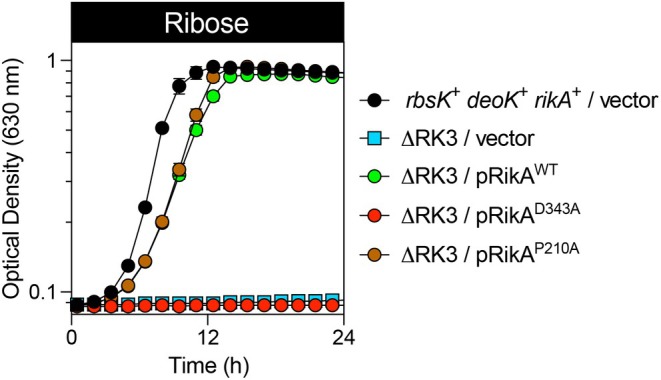
Residue D343 of RikA is the active site residue. Each strain was grown overnight in NB + ampicillin (100 μg/mL) at 37°C with agitation and sub‐cultured 1:100 into NCE minimal medium with ribose (22 mM) as the sole source of carbon and energy. Cultures were grown in 96‐well plates at 37°C with agitation. ∆RK3 stands for the ∆*rbsK84* ∆*deoK408 rikA::cat* ^+^ triple deletion, ‘Vector’ stands for the empty cloning vector pCV1 that contains an arabinose‐inducible promoter, pRikA^WT^ is pRikA‐2, pRikA^D343A^ is pRikA‐6, pRikA^P210A^ is pRikA‐7. Plasmids were maintained with ampicillin (100 μg/mL), and ectopic gene expression was induced with L‐(+)‐arabinose (500 μM). This experiment was conducted in technical triplicate of biological duplicates. Error bars represent one standard deviation from the mean. Error bars that are not visible are smaller than the symbol.

To determine whether residue D343 was required for RikA function, we constructed complementation plasmids carrying missense mutations D343A and P210A. Residue P210 was chosen as a control because it is conserved in the proteins analyzed here but has not been reported to be involved in the enzymatic function or ligand binding in *E. coli* RbsK. We assayed growth on ribose of the ∆RK3 strain ectopically producing either RikA^D343A^ or RikA^P210A^ to determine the effect of each mutation. The ∆RK3 strain expressing RikA^P210A^ grew like a strain producing the wildtype RikA protein (Figure 5 brown circles vs green circles), while the strain expressing RikA^D343A^ failed to grow (Figure 5, red circles). These results suggested that residue D343 in RikA is required for protein function, although this experiment cannot rule out the possibility that the D343A substitution interferes with proper protein folding or solubility.

### 2.3 Kinetic Analysis of Ribose Kinases From *S* . Typhimurium

**FIGURE 6:**
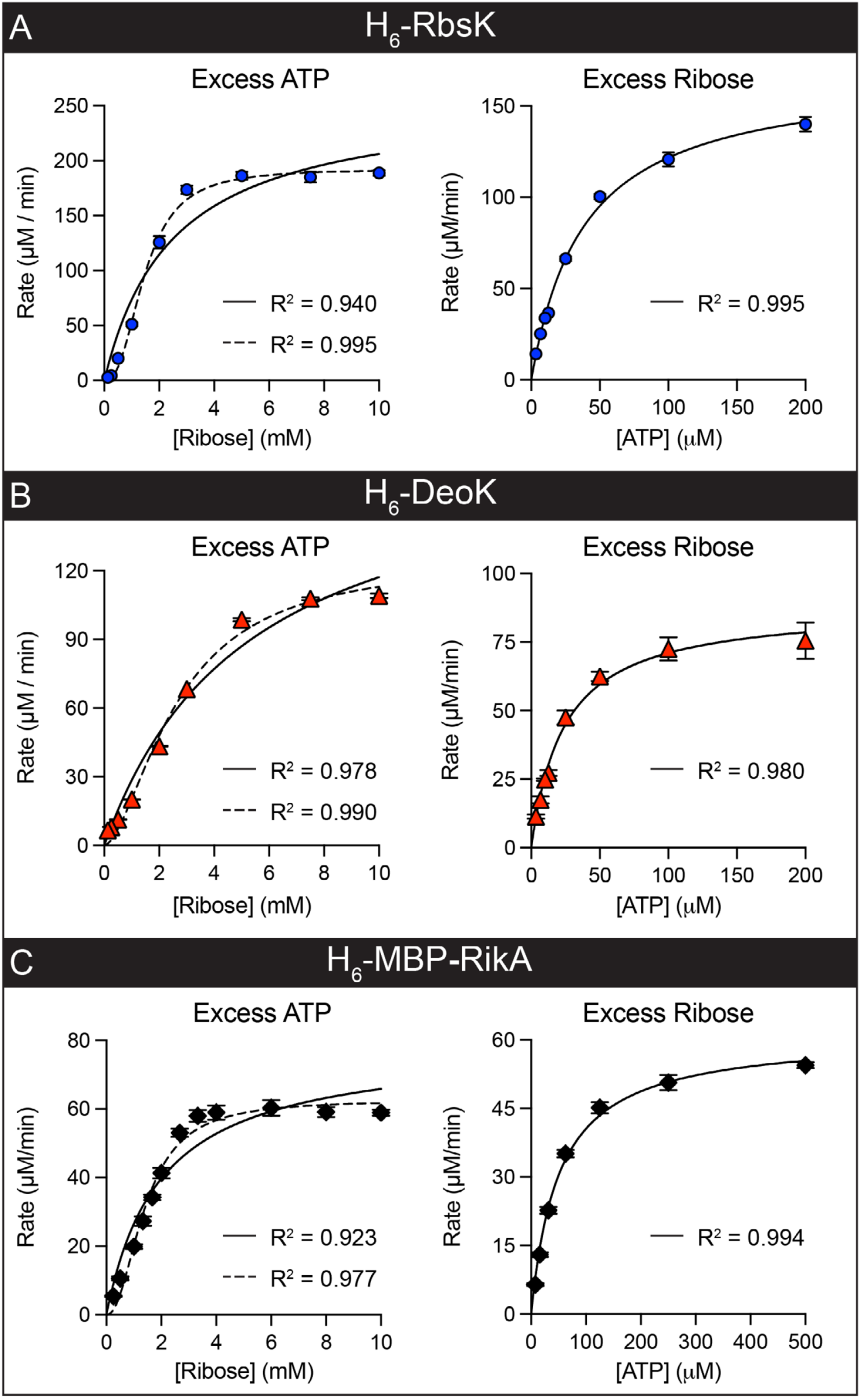
Saturation curves for ribose kinase activity by RbsK, DeoK, and RikA from *S.* Tyhpimurium. Ribose kinase activity for each enzyme was determined with excess ATP (left panels) and excess ribose (right panels). Each point is the mean of three technical replicates, and error bars represent one standard deviation from the mean. Solid lines are Michaelis–Menten curves, and dashed lines are Hill curves (left panels only). Error bars that are not visible are smaller than the symbol.

To assess the kinetics of the reactions catalyzed by each ribokinase, we purified RbsK, DeoK, and RikA, each to ≥ 85% homogeneity (Figure S3). RbsK and DeoK proteins were overproduced using plasmid pTEV18 (VanDrisse and Escalante‐Semerena 2016), which encoded N‐terminal hexahistidine (H_6_) tagged proteins, whose tags were not removed. H_6_‐RikA was not in the soluble fraction when overexpressed from plasmid pTEV18, so it was produced using plasmid pTEV19, which encoded an N‐terminal hexahistidine‐maltose binding protein (H_6_‐MBP) tagged protein (VanDrisse and Escalante‐Semerena 2016). The tag could not be removed from H_6_‐MBP‐RikA by rTEV protease, but it did not interfere with the physiological function of the protein (Figure S4). Pseudo first order kinetics was performed under saturation for either ribose or ATP. Apparent kinetic parameters were determined and are reported in Figure 6 and Tables 1 and 2.

**TABLE 1:** Michaelis–Menten analysis of ribose kinase kinetics of RbsK, DeoK, and RikA from *S.* Typhimurium.

Reaction substrates	*V*_*max*_ (95% confidence)	*K*_*m*_ (95% confidence)	*k*_*cat*_ (95% confidence)	*k*_*cat*_/*K*_*m*_
	μM NADH consumed/min		min^−1^	min^−1^ μM^−1^
RbsK				
Ribose	257.7 (227.4–296.2)	2.5 mM (1.8–3.6 mM)	27,828 (24,558–31,987)	11.2
ATP	168.5 (163.1–174.3)	38.5 μM (35.2–42.0 μM)	18,200 (17,610–18,821)	473.3
DeoK				
Ribose	179.3 (158.9–206.2)	5.3 mM (4.1–7.0 mM)	458.8 (406.6–527.5)	0.1
ATP	88.0 (83.5–92.8)	23.8 μM (20.5–27.8 μM)	225.1 (213.7–237.3)	9.4
RikA				
Ribose	78.8 (72.0–86.7)	2.0 mM (1.54–2.53 mM)	337.4 (308.3–370.9)	0.2
ATP	61.1 (60.2–61.9)	50.5 μM (46.9–54.3 μM)	261.3 (257.6–265.1)	5.2

*Note:* Kinetics of each purified enzyme were assessed for ribose and ATP. Each substrate was analyzed while the other was in excess. Values were calculated in Prism v10 (GraphPad) from 3 replicates.

**TABLE 2:** Hill equation analysis of ribose kinase kinetics of RbsK, DeoK, and RikA from *S.* Typhimurium with ATP in excess.

Protein	*V*_*max*_ (95% confidence)	*K*_*A*_ (95% confidence)	Hill coefficient (95% confidence)
	μM NADH consumed/min	mM	
H_6_‐RbsK	192.6 (187.6–197.8)	1.5 (1.4–1.6)	2.5 (2.2–2.8)
H_6_‐DeoK	125.3 (116.3–138.7)	2.7 (2.4–3.2)	1.7 (1.4–2.0)
H_6_‐MBP‐RikA	62.6 (60.2–65.4)	1.4 (1.3–1.5)	2.2 (1.8–2.6)

*Note:* Using the Hill Equation, sigmoid kinetic analysis of each purified enzyme was performed on data with ATP in excess and variable concentrations of ribose. Values were calculated in Prism v10 (GraphPad) from three replicates.

H_6_‐MBP‐RikA exhibited similar *K*_*m*_ values for ribose as H_6_‐RbsK and H_6_‐DeoK (2.0, 2.5, and 5.3 mM, respectively) and ATP (50.5, 38.5, and 23.8 μM, respectively). H_6_‐RbsK, however, displayed *K*_*cat*_ and *K*_*cat*_/*K*_*m*_ values that were two orders of magnitude higher than H_6_‐DeoK and H_6_‐MBP‐RikA for both substrates, and H_6_‐MBP‐RikA had a *V*_*max*_ 4 and 2 times lower than H_6_‐RbsK and H_6_‐DeoK, respectively (Table 1). Each enzyme exhibited Michaelis–Menten kinetics with regards to ATP (Figure 6, right panels), but we noticed that the saturation curves for ribose appeared sigmoidal (Figure 6, left panels). We analyzed the ribose saturation curves for each H_6_‐RbsK, H_6_‐DeoK, and H_6_‐MBP‐RikA using the Hill Equation (Hill 1910) and found that the Hill curve fit our data more closely than the Michaelis–Menten curve (dashed vs. solid lines, see *R* ^2^ values, Figure 6, left panels). These analyses also found that the Hill coefficient was greater than one for each protein (Table 2), suggesting that ribose binding by H_6_‐RbsK, H_6_‐DeoK, and H_6_‐MBP‐RikA increased their affinity for ATP. Crystallographic investigation of *E. coli* RbsK found that ribose binding induced conformational changes in the ATP binding pocket, likely making ATP binding more favorable (Sigrell et al. 1999). Based on the data obtained in these studies we suggest that this mechanism is conserved in *S. typhimurium* RbsK and present in DeoK and RikA.

### 2.4 Pentoses Other Than Ribose Are Poor Substrates for the RikA Kinase

We tested whether RikA could phosphorylate pentoses other than ribose. For this purpose, we purified H_6_‐MBP‐RikA^D343A^ as a negative control for these reactions. Although we isolated H_6_‐MBP‐RikA^D343A^ protein, the fraction containing it had substantially more contamination than the fraction containing H_6_‐MBP‐RikA^WT^ protein (Figure S3). To account for this contamination, BSA was added to reaction mixtures containing H_6_‐MBP‐RikA^WT^ protein to simulate the contamination observed in preparations of H_6_‐MBP‐RikA^D343A^ protein. We found that the addition of BSA did not affect the rate of the reaction catalyzed by H_6_‐MBP‐RikA^WT^ on ribose. Additionally, the H_6_‐MBP‐RikA^D343A^ variant had no ribose kinase activity (Table 3), supporting our earlier conclusion that D343 of RikA is critical for activity. H_6_‐MBP‐RikA^WT^ phosphorylated D‐deoxyribose at a rate about 40 times slower than ribose (Table 3), consistent with our observation that RikA did not support growth on deoxyribose (Figure 3C,D). We also found that H_6_‐MBP‐RikA^WT^ had detectable kinase activity with D‐xylose and D‐xylulose, but the rate was two orders of magnitude lower than the rate measured with ribose, and we detected no activity with L‐arabinose as the substrate (Table 3).

**TABLE 3:** RikA has little to no activity on other common pentoses.

Substrate	H_6_‐MBP‐RikA^WT^	H_6_‐MBP‐RikA^D343A^
	Reaction rate (μM/min) (95% confidence)	
D‐Ribose	80.7 (79.6–81.8)	0.825 (−0.4–2.0)
D‐Deoxyribose	2.3 (2.2–2.3)	0.377 (0.4–0.4)
L‐Arabinose	0.319 (0.3–0.3)	0.322 (0.3–0.3)
D‐Xylose	0.651 (0.6–0.7)	0.453 (0.4–0.4)
D‐Xylulose	0.771 (0.7–0.8)	0.532 (0.5–0.6)

*Note:* The ability of H_6_‐MBP‐RikA^WT^ to phosphorylate pentoses was tested using the assay described previously. The concentration of each pentose was 10 mM and ATP was 2 μM.

### 2.5 RikA Appears to Begin at the Methionine Annotated at Position 90

We noticed in the multiple sequence alignment that RikA appears to have a 90‐residue N‐terminal extension relative to *S.* Typhimurium DeoK and RbsK from both *S.* Typhimurium and *E. coli* (Figure 4). Further analysis using PROSITE predicted that residues 2–57 of RikA form a DeoR family DNA binding domain (Sigrist et al. 2026). Since RikA contains a methionine at position 90 that closely aligns with the starter methionine for *S.* Typhimurium DeoK and RbsK from *S.* Typhimurium and *E. coli* (Figure 4), we decided to test whether the first 89 amino acids of RikA were present in the protein synthesized under our growth conditions. For this purpose, we cloned 278 base pairs upstream of the start codon, the predicted coding sequence of *rikA*, and the required sequence to produce a C‐terminal triple glycine–alanine hexahistidine [(GA)_3_‐H_6_] epitope into plasmid pCV2. Plasmid pCV2 contains a P_*araBAD*_ promoter responsive to L‐Ara but does not encode a ribosome binding site (RBS), so any protein produced from the plasmid will be translated from the native RBS.

**FIGURE 7:**
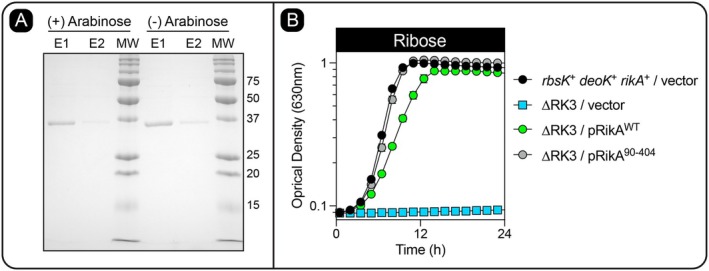
RikA appears to begin at methionine 90. (A) The ∆RK3/pRikA‐9 (RikA^90‐404^‐(GA)_3_‐H_6_) strain was grown overnight in NB + ampicillin (100 μg/mL) at 37°C with agitation and sub‐cultured 1:100 into 25 mL NCE minimal medium with ribose (22 mM) as the sole source of carbon and energy, either with or without arabinose (500 μM). Cultures were grown at 37°C with agitation for 18 h, and H_6_‐tagged protein was purified from the cells as described in *Experimental procedures*. SDS‐PAGE analysis revealed a band with mass of ~35 kDa in both the (+) and (−) arabinose conditions. MW is the molecular weight ladder (Precision Plus, Bio‐Rad), and numbers to the right of the image are the masses of each band in the ladder. (B) Each strain was grown overnight in NB + ampicillin (100 μg/mL) at 37°C with agitation and sub‐cultured 1:100 into NCE minimal medium with ribose (22 mM) as the sole source of carbon and energy. Cultures were grown in 96‐well plates at 37°C with agitation. ∆RK3 stands for the ∆*rbsK84* ∆*deoK408 rikA::cat* ^+^ triple deletion, ‘Vector’ stands for the empty cloning vector pCV1 that contains an arabinose‐inducible promoter, pRikA^WT^ is pRikA‐2, pRikA^90‐404^ is pRikA‐10. Plasmids were maintained with ampicillin (100 μg/mL), and ectopic gene expression was induced with L‐(+)‐arabinose (500 μM). This experiment was conducted in technical triplicate of biological duplicates. Error bars represent one standard deviation from the mean. Error bars that are not visible are smaller than the symbol.

We transformed the resulting plasmid (pRikA9, see 4. *Experimental procedures*) into the ∆RK3 strain and grew the resulting strain in ribose minimal medium with or without arabinose. Both cultures grew to full density, and any H_6_‐tagged protein was purified from each culture using NEBExpress Ni Spin columns and examined by SDS‐PAGE. We recovered a pure protein from each culture with a molecular mass of ~35 kDa, which is what we would expect if the protein started at the predicted M90 (34.79 kDa including the (GA)_3_‐H_6_ tag) instead of the annotated M1 (44.67 kDa including the (GA)_3_‐H_6_ tag) (Figure 7A). We excised the ~35 kDa bands and submitted them to the University of Georgia Proteomics and Mass Spectrometry core facility for analysis. The bands recovered from cells grown both with and without arabinose were identified as belonging to RikA‐(GA)_3_‐H_6_, and mass spectra and peak lists are presented in Figure S5.

To directly test whether RikA^90‐404^ could support growth on ribose, we expressed it from plasmid pCV1 in the ∆RK3 strain and assayed growth in ribose minimal medium as described under *Experimental procedures*. We found that the ∆RK3/pRikA^90‐404^ strain grew as well as the *rbsK* ^+^ *deoK* ^+^ *rikA* ^+^ strain when using ribose as the sole carbon and energy source (Figure 7B, gray circles vs. black circles). Together, these data suggested that RikA^90‐404^ was the functional form of the protein synthesized under our growth conditions, although we cannot rule out the synthesis of the putative RikA isoform that contains residues 1–90 under other environmental conditions.

### 2.6 RikA and Its Genetic Neighborhood Are Conserved in a Subset of Entobacterales

**FIGURE 8:**
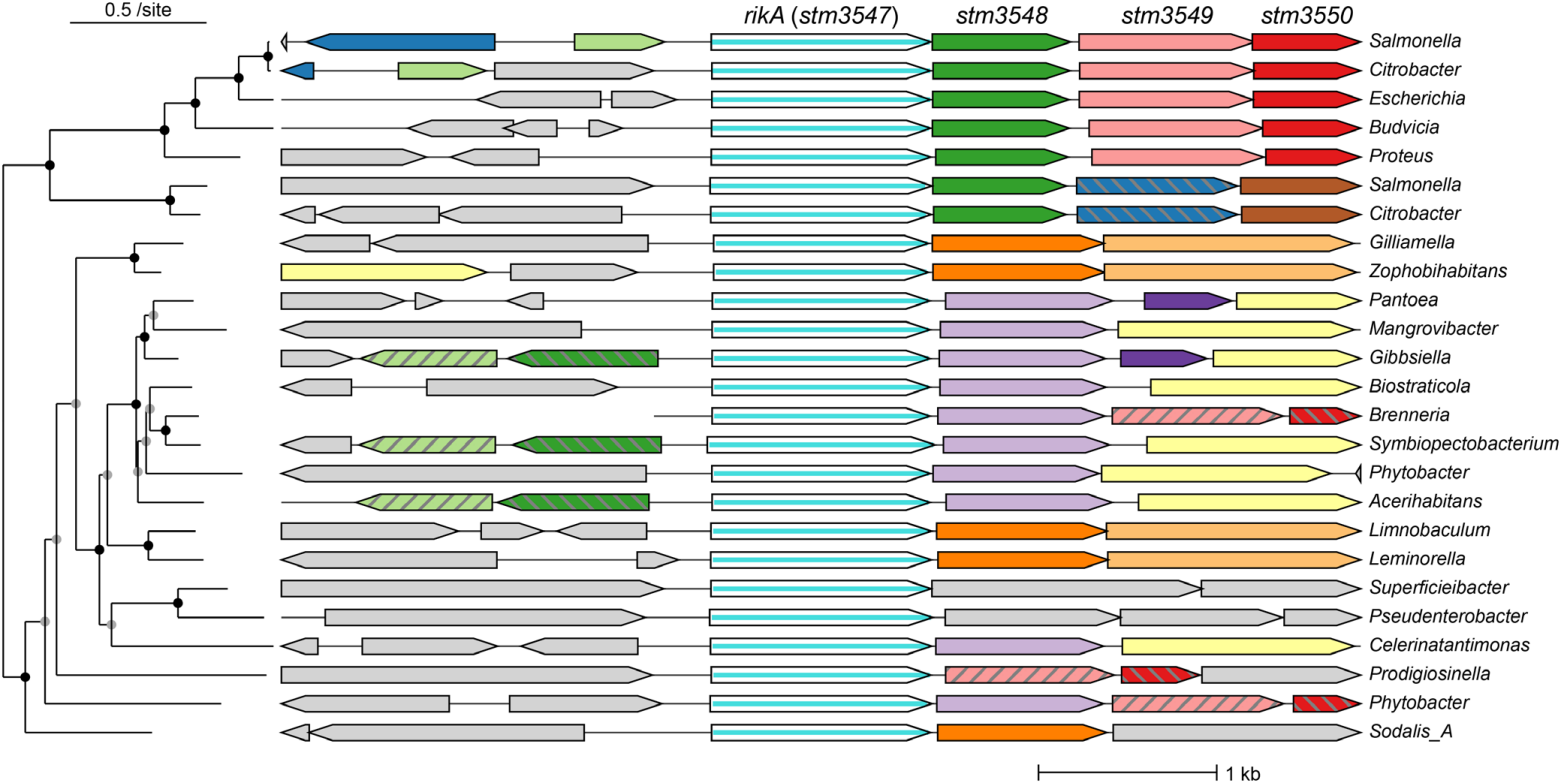
The *rikA* gene and three genes downstream are conserved in a subset of Enterobacterales. RikA was analyzed using the browser‐based tool, *fast.genomics* against the representative bacterial and archaeal genome database maintained by *fast.genomics* (Price and Arkin 2024). Gene neighborhoods of the top 25 hits are displayed here. Homologs of *rikA* are in blue and white, *stm3548* and its homologs are in green, *stm3549* and its homologs are in pink, and *stm3550* and its homologs are in red.

To assess the conservation and distribution of RikA, we analyzed the protein sequence using the browser tool *fast.genomics* (Price and Arkin 2024) using the “gene neighborhoods” option. We found that the gene cluster from *rikA* (*STM3547*) to *stm3550* is conserved in representative species of the *Citrobacter*, *Escherichia*, *Budvicia*, and *Proteus* genera (Figure 8). We have not found studies into the function(s) of STM3548, STM3549, STM3550, or their homologs found here, although a crystal structure of STM3548 has been published (Petrova et al. 2007). The predicted functions of these proteins and their homologs, however, may shed some light on the function of this genetic locus. The STM3548 homolog in *Citrobacter* is predicted to be a glutamine amidotransferase, the STM3549 homolog is annotated as a DMT family transporter, and STM3550 is a putative phosphotriesterase. These four proteins (including RikA) may constitute a metabolic pathway and transporter for an unknown carbon source, and the ribose kinase activity of RikA may be off‐target activity as is the case with DeoK. The lower branch of the phylogenetic tree produced by this analysis suggests that there are homologs to *rikA* in a broad range of bacterial genera (Figure 8, *Gilliamella* and lower). The genetic context of these *rikA* homologs, however, is different from that found in *S.* Typhimurium, *Citrobacter*, *Escherichia*, *Budvicia*, and *Proteus*, and proteins from *Gilliamella* and lower on the image are on the earliest diverging branch of the phylogenetic tree generated here. From these data, it is difficult to determine whether the RikA homologs in the lower branch are true homologs of RikA or are different members of the PfkB carbohydrate kinase family.

## 3 Conclusions

In the present work, we have shown that RikA (STM3547) from *S. typhimurium* is a functional ribose kinase and a member of the PfkB family of carbohydrate kinases. Kinetic analysis of RikA with ribose and ATP found that RikA exhibits *K*_*cat*_ and *K*_*cat*_/*K*_*m*_ values an order of magnitude lower than RbsK from *S.* Typhimurium and like *S.* Typhimurium DeoK, with respect to both substrates. Additionally, we demonstrated that ribose binding increased the affinity of each protein tested for ATP, in accordance with earlier observations of *E. coli* RbsK (Sigrell et al. 1999). The annotated amino acid sequence of RikA appears to have a 90‐residue N‐terminal extension relative to RbsK and DeoK, and this region is predicted to encode a DeoR‐type DNA binding domain. We have also presented evidence that the putative 89‐residue N‐terminal extension is not present in the RikA protein synthesized under our growth conditions, and that RikA may start at the methionine annotated at position 90. RikA^90‐404^ can support growth on ribose as the sole carbon and energy source, and RikA aligns more closely to other members of the PfkB family (Figure 4; Wu et al. 1991) if methionine at position 90 is the start codon. More work is needed, however, to verify this conclusion. Finally, we found that *rikA* and the three genes downstream (*stm3548*, *stm3549*, and *stm3550*) are conserved in a subset of Enterobacterales. Little is known about these genes and their homologs; however, they are predicted to be a glutamine amidotransferase, DMT family transporter, and phosphotriesterase, respectively. It is possible that these four genes encode a metabolic pathway for an unknown substrate, and that the ribose kinase activity of RikA is a moonlighting function.

## 4 Experimental Procedures

### 4.1 Bacterial Strains and Growth Conditions

All strains in this study are described in Table 4. All experimental strains used in this study are derivatives of *Salmonella enterica* subsp. *enterica* sv. Typhimurium str. LT2 ( *S.* Typhimurium). The *rikA* deletions were constructed using the technique described by Datsenko and Wanner (2000). The insertion cassettes from pKD3 and pKD4 were PCR amplified using Phusion High‐Fidelity DNA polymerase (ThermoFisher) and verified by agarose gel electrophoresis with ethidium bromide staining. The forward and reverse primers had 39 base pair 5′ overhangs with homology to the start and end of *rikA*, respectively. Products were cleaned with the Wizard SV gel and PCR clean up kit (Promega), and ~1 μg of PCR product was electroporated into electrocompetent *S.* Typhimurium strain JE6692 using a 0.2‐cm electroporation cuvette (MidiSci) and a microPulser electroporator (Bio‐Rad Laboratories) on Ec2 setting. Cells were recovered at 37°C with shaking and plated on LB (Difco) agar containing 20 μg/mL chloramphenicol for the *cat* ^+^ marker or 50 μg/mL kanamycin for the *kan* ^+^ marker. Correct insertions were verified by PCR and moved into the necessary strain(s) by P22‐mediated transduction (Davis et al. 1980). The *rbsK83::kan* ^+^ and *deoK407::kan* ^+^ deletions were moved from the BEI Single Gene Deletion collection (Porwollik et al. 2014) into strain JE6583 and its descendant strains as needed by P22‐mediated transduction. Antibiotic resistance cassettes were removed as needed using the helper plasmid, pCP20 (Datsenko and Wanner 2000), yielding the ∆*rbsK84* and ∆*deoK408* alleles, respectively. Routine culturing was performed on lysogenic broth (LB) agar (1.5%) or broth supplemented with the appropriate antibiotic(s) at 37°C, with shaking for liquid cultures.

**TABLE 4:** Strains used in this study.

Strain	Relevant genotype	Reference/Source^a^
***S*** . **Typhimurium** ^**b**^ **strains**		
JE6583	*metE205* ∆*araB9*	K. Sanderson via J. Roth
BEI 4997	*S.* Typhimurium str. 14028s^c^ *rbsK::kan* ^+^	From BEI Collection (SGD 156/157 Kan, F12)^d^ (Porwollik et al. 2014)
BEI 4879	*S.* Typhimurium str. 14028s^c^ *deoK::kan* ^+^	From BEI Collection (SGD 015/016 Kan, C4)^d^ (Porwollik et al. 2014)
*Derivatives of strain JE6583*		
JE27669	*rbsK83::kan* ^+^	
JE27670	*deoK407::kan* ^+^	
JE28123	*rikA::cat* ^+^	
JE28372	*rikA::kan* ^+^	
JE28124	∆*rbsK84* ∆*deoK408*	
JE28151	∆*rbsK84 rikA::cat* ^+^	
JE28134	*deoK407::kan* ^+^ *rikA::cat* ^+^	
JE28318	∆*rbsK84* ∆*deoK408 rikA::cat* ^+^	
JE22070	/pCV1 (cloning vector) *bla* ^+^	VanDrisse and Escalante‐Semerena (2016)
JE27687	*rbsK83::kan* ^+^/pCV1 *bla* ^+^	
JE28386	*rikA::kan* ^+^/pCV1 *bla* ^+^	
JE28186	*deoK407::kan* ^+^/pCV1 *bla* ^+^	
JE28187	∆*rbsK84* ∆*deoK408*/pCV1 *bla* ^+^	
JE28188	∆*rbsK84 rikA::cat* ^+^/pCV1 *bla* ^+^	
JE28189	*deoK407::kan* ^+^ *rikA::cat* ^+^/pCV1 *bla* ^+^	
JE28396	∆*rbsK84* ∆*deoK408 rikA::cat* ^+^/pCV1 *bla* ^+^	
JE28397	∆*rbsK84* ∆*deoK408 rikA::cat* ^+^/pRikA‐2 *bla* ^+^ *rikA* ^+^	
JE28420	∆*rbsK84* ∆*deoK408 rikA::cat* ^+^/pRbsK‐1 *bla* ^+^ *rbsK* ^+^	
JE28516	∆*rbsK84* ∆*deoK408 rikA::cat* ^+^/pDeoK‐1 *bla* ^+^ *deoK* ^+^	
JE28646	∆*rbsK84* ∆*deoK408 rikA::cat* ^+^/pRikA‐4 *bla* ^+^ Encodes H_6_‐MBP‐RikA^WT^	
JE28707	∆*rbsK84* ∆*deoK408 rikA::cat* ^+^/pRikA‐6 *bla* ^+^ Encodes RikA^D343A^	
JE28725	∆*rbsK84* ∆*deoK408 rikA::cat* ^+^/pRikA‐7 *bla* ^+^ Encodes RikA^P210A^	
JE28887	∆*rbsK84* ∆*deoK408 rikA::cat* ^+^/pRikA‐9 *bla* ^+^, encodes the entire coding sequence of *rikA* ^WT^ and 278 bp upstream of the predicted start codon. RikA is expressed with a C‐terminal (GA)_3_‐H_6_ tag	
JE28939	∆*rbsK84* ∆*deoK408 rikA::cat* ^+^/pRikA‐10 *bla* ^+^ *rikA* ^+^, encodes RikA^90‐404^	
JE6692	/ pKD46 *bla* ^+^	Laboratory collection
***E. coli*** **strains**		
*E. coli* BL21 (λDE3)	F^−^ *ompT gal dcm lon hsdS*_*B*_ (*r*_*B*_‐ *m*_*B*_‐) λ(*DE3* [*lacI lacUV5*‐*T7p07 ind1 sam7 nin5*]) [*malB* ^+^]_K‐12_(λ^*S*^)	Laboratory collection
*E. coli* DH5⍺	∆*(argF‐lacZ)169 ∆phoA8 l* ^*−*^f80d*lacZ58*(M15) *thiE1* *glnX44*(AS) *deoR481 hsdR17* *gyrA96*(Nal^R^) *recA1 endA1*	Laboratory collection

^a^ Unless otherwise stated, all strains were constructed during this work.

^b^ *S. typhimurium* is an abbreviation of *Salmonella enterica* subspecies *enterica* serovar Typhimurium strain LT2. Other strains are noted where applicable.

^c^ *S. typhimurium* str. 14,028 s is an abbreviation of *Salmonella enterica* subspecies *enterica* serovar Typhimurium strain 14028s.

^d^ The indicated strains were obtained through BEI Resources, NIAID, NIH: *Salmonella enterica* subsp. *enterica* strain 14028s (serovar Typhimurium) Single Gene Deletion Mutant Library.

#### 4.1.1 Plasmids and Plasmid Construction

The high‐efficiency method utilizing BspQI (Galloway et al. 2013; VanDrisse and Escalante‐Semerena 2016) was used to clone *rbsK*, *deoK*, and *rikA* into plasmid pCV1, *rbsK* and *deoK* into plasmid pTEV18, and *rikA* into plasmid pTEV19. Each gene was amplified from *S. typhimurium* strain JE6583 genomic DNA using Phusion High‐Fidelity DNA polymerase with primers containing BspQI cleavage sites and vector‐compatible overhangs. Products were verified and cleaned as described above. Cloning reaction products were transformed into *E. coli* DH5α, colonies were screened by colony PCR, and plasmids were isolated using the Wizard Plus SV Miniprep kit (Promega). Sanger sequencing was performed by Eton Biosciences to confirm the insertions. The plasmid pRikA‐4 (Table 5) was constructed by PCR amplifying the H_6_‐MBP‐*rikA* construct from pRikA‐3 using primers RDM283 and AAJ48 (Table 6). This product was verified, cleaned, and cloned into pCV1 using the method described above.

**TABLE 5:** Plasmids used in this study.

Plasmid	Genotype	Description	Source/Reference^a^
pCV1	*E. coli araC* ^+^ *bla* ^+^	P_*araBAD*_ expression vector	VanDrisse and Escalante‐Semerena (2016)
pCV2	*E. coli araC* ^+^ *bla* ^+^	P_*araBAD*_ expression vector, does not encode a ribosome binding site	VanDrisse and Escalante‐Semerena (2016)
pTEV18	*bla* ^+^	Overexpression vector for H_6_‐N‐terminally tagged rTEV‐cleavable protein	VanDrisse and Escalante‐Semerena (2016)
pTEV19	*bla* ^+^	Overexpression vector for H_6_‐MBP‐N‐terminally tagged rTEV‐cleavable protein	VanDrisse and Escalante‐Semerena (2016)
pRbsK‐1	*rbsK* ^+^ *bla* ^+^	*rbsK* ^+^ cloned into pCV1	
pRbsK‐3	*rbsK* ^+^ *bla* ^+^	Overexpression of H_6_‐RbsK, pTEV18 backbone	
pDeoK‐1	*deoK* ^+^ *bla* ^+^	*deoK* ^+^ cloned into pCV1	
pDeoK‐3	*deoK* ^+^ *bla* ^+^	Overexpression of H_6_‐DeoK, pTEV18 backbone	
pRikA‐2	*rikA* ^+^ *bla* ^+^	*rikA* ^+^ cloned into pCV1	
pRikA‐3	*rikA* ^+^ *bla* ^+^	Overproduction of H_6_‐MBP‐RikA, pTEV19 backbone	
pRikA‐4	*rikA* ^+^ *bla* ^+^	H_6_‐MBP‐RikA cloned into pCV1	
pRikA‐5	*rikA* ^+^ *bla* ^+^	Overproduction of H_6_‐MBP‐RikA^D343A^, pTEV19 backbone	
pRikA‐6	*rikA* ^+^ *bla* ^+^	RikA^D343A^ cloned into pCV1	
pRikA‐7	*rikA* ^+^ *bla* ^+^	RikA^P210A^ cloned into pCV1	
pRikA‐9	*rikA* ^+^ *bla* ^+^	RikA‐(GA)_3_‐H_6_ and 278 base pairs upstream of the predicted start codon cloned into pCV2	
pRikA‐10	*rikA* ^+^ *bla* ^+^	*rikA* ^+^ cloned into pCV1, encodes RikA^90‐404^	
pCP20	*flp* ^+^ *bla* ^+^ *cat* ^+^	FLP expression plasmid, temperature sensitive above 37°C	Cherepanov and Wackernagel (1995)

^a^ Unless otherwise stated, all plasmids were engineered during this work.

**TABLE 6:** Primers used in this study.^a^

Name	Purpose	Sequence (5′–3′)^b^
AAJ27	Cloning *rbsK* into pCV1 and pTEV18, forward	NNGCTCTTCNTTCATGAAAACCGCAGGTAATCTC
AAJ28	Cloning *rbsK* into pCV1 and pTEV18, reverse	NNGCTCTTCNTTATTACCCCTGCTGATGTAAAAACGC
RDM252	Cloning *deoK* into pCV1 and pTEV18, forward	NNGCTCTTCNTTCATGGATATCGCGGTTATTGG
RDM253	Cloning *deoK* into pCV1 and pTEV18, reverse	NNGCTCTTCNTTATTATTCGTTCAACGAAAGATACTC
AAJ47	Cloning *rikA* into pCV1 and pTEV19, forward	NNGCTCTTCNTTCATGAAATTTGAACGCCATCATAG
AAJ48	Cloning *rikA* into pCV1 and pTEV19, reverse	NNGCTCTTCNTTATTAGTGAGTAGAGATAGTTTGTTTG
AAJ43	Amplification of pKD3 and pKD4 antibiotic resistance cassettes, for deletion of *rikA*, forward	ATGAAATTTGAACGCCATCATAGAATATTGAAGGAACTCGTGTAGGCTGGAGCTGCTTC
AAJ44	Amplification of pKD3 and pKD4 antibiotic resistance cassettes, for deletion of *rikA*, reverse	TTAGTGAGTAGAGATAGTTTGTTTGTAATGTGAACCCTGCATATGAATATCCTCCTTAG
RDM284	Missense mutation to produce variant RikA^D343A^	GGGAGCGGGTGCCGCTTTCAATGGTG
RDM285	Missense mutation to produce variant RikA^D343A^	CATTGAAAGCGGCACCCGCTCCCGCC
RDM286	Missense mutation to produce variant RikA^P210A^	GAATGGTGATTTCATCAGCGGAAATTGTCATGTTGGC
RDM287	Missense mutation to produce variant RikA^P210A^	GCCAACATGACAATTTCCGCTGATGAAATCACCATTC
RDM283	Cloning H_6_‐MBP‐RikA into pCV1 (with AAJ48), forward	NNGCTCTTCNTTCATGGGCAGCCATCACCATC
RDM290	Cloning *rikA* and the 278 basepairs upstream of the predicted start codon into pCV2, forward	NNGCTCTTCNTTCCGCGCGTGAAATAACGCTTC
RDM291	Cloning *rikA* into pCV2, contains overhangs to add a (GA)_3_‐H_6_ C‐terminal tag to RikA, reverse 1	GTGATGATGGTGATGATGTGCTCCAGCTCCTGCACCGTGAGTAGAGATAGTTTGTTTG
RDM292	Cloning *rikA* into pCV2, contains overhangs to add a BspQI site to the (GA)_3_‐H_6_ C‐terminal tagged RikA, reverse 2	NNGCTCTTCNTTAGTGATGATGGTGATGATGTG
RDM293	Cloning *rikA* into pCV1 (with AAJ48). Resulting plasmid encodes RikA^90‐404^, forward	NNNNNNAAGCTTCGCTCTCCTGTAGCGTGAT

^a^ All primers were synthesized by Integrated DNA Technologies (IDT, Coralville, IA).

^b^ Where *N* can be any deoxynucleotide.

#### 4.1.2 Site‐Directed Mutagenesis

Missense mutations were made using the QuikChange method (Stratagene). Primers RDM284 and RDM285 and Pfu Ultra II DNA Polymerase were used to PCR amplify pRikA‐3 (Table 5) with extension at 68°C for 2.5 min per kilobase and introduce the D343A mutation. The product of this reaction was digested by DpnI for 6 h at 37°C to remove any template plasmid, then transformed into *E. coli* DH5⍺. Cultures were grown overnight from single colonies, and plasmids were purified using the Wizard Plus SV Minipreps kit (Promega). The sequence of the gene encoding the RikA^D343A^ variant was confirmed by Sanger sequencing (Eton Biosciences), and the plasmid was named pRikA‐5. The *rikA* allele encoding the RikA^D343A^ variant was PCR amplified from pRikA‐5 using primers AAJ47 and AAJ48. This product was verified, cleaned, and cloned into pCV1 using the method described above resulting in plasmid pRikA‐6. Primers RDM286 and RDM287 were used to amplify pRikA‐2 and introduce the P210A change (pRikA‐7) as described above for pRikA‐5.

#### 4.1.3 Growth Analyses

Strains were streaked to isolation on LB agar with ampicillin (100 μg/mL) and grown overnight at 37°C. Single colonies were inoculated into nutrient broth (NB, Difco) with 100 μg/mL ampicillin and grown 18–20 h at 37°C with shaking at 180 rpm. Overnight cultures were sub‐cultured 1:100 into 200 μL of no‐carbon essential (NCE) medium (Berkowitz et al. 1968) supplemented with MgSO_4_ (1 mM), Wolfe's trace minerals (Balch and Wolfe 1976), cyanocobalamin (15 nM), ampicillin (100 μg/mL), L‐(+)‐arabinose (concentration indicated in figure legends) and carbon source(s) as detailed in the text and figure legends. Growth studies were performed in 96‐well polystyrene (Falcon) microtiter plates. Plates were incubated at 37°C shaking continuously inside an 800TS, Eon, or ELx808 microtiter plate reader (Bio‐Tek Instruments). Density of cells was monitored at 630 nm and data were analyzed using Prism v10 (GraphPad).

#### 4.1.4 Alignment, Visualization, and Analysis of Protein Sequences

Amino acid sequences were obtained from Biocyc.org for the RbsK, DeoK (STM3793), and RikA (STM3547) proteins from *S.* Typhimurium, and RbsK from *E. coli* K‐12 substr. MG1655. The sequences were aligned using Clustal Omega (Madeira et al. 2024) with default settings. The alignment was visualized using ESPript 3.0 with default settings and Flashy color scheme (Robert and Gouet 2014). Amino acid functional annotations were obtained from the UniProt entry for *E. coli* K12 RbsK (P0A9J6), and PfkB‐family signature patterns (P00583 and P00584) were obtained from the PROSITE database (Sigrist et al. 2026).

#### 4.1.5 Overproduction of Proteins Used in This Work

Proteins H_6_‐RbsK, H_6_‐DeoK, H_6_‐MBP‐RikA^WT^, and H_6_‐MBP‐RikA^D343A^ were produced from the plasmids pRbsK‐3, pDeoK‐3, pRikA‐3, and pRikA‐7, respectively. Each plasmid was separately transformed into *E. coli* BL21(λDE3). A single colony was chosen from each transformation plate and grown overnight in LB (20 mL) with ampicillin (100 μg/mL) at 37°C shaking at 180 rpm. For the pRbsK‐3 and pDeoK‐3 cultures, 10 mL of the overnight culture was sub‐cultured into LB (250 mL) plus ampicillin (100 μg/mL). Cultures were grown at 37°C with shaking at 180 rpm until the optical density (OD) was ~0.5, isopropyl β‐D‐1‐thiogalactopyranoside (IPTG) was added to a concentration of 250 μM to induce gene expression from the plasmids, and cells were returned to 37°C 180 rpm incubation for 4 h. Cells were harvested by centrifugation at 6000 × *g* for 15 min using a refrigerated Beckman Coulter Avanti J‐20‐XPI centrifuge equipped with a JLA 8.1 rotor. Pellets were stored @ −20°C until used.

For the pRikA‐3 and pRikA‐7 cultures, 20 mL of the overnight culture was sub‐cultured into 1 L super optimal broth with catabolite repression (SOC) (Hanahan 1983). The culture was grown at 37°C with shaking at 180 rpm to an optical density (OD) of ~0.5; gene expression was induced by IPTG at 250 μM, and cells were incubated at 20°C with shaking at 180 rpm overnight. Cells were harvested by centrifugation at 6000 × *g* for 15 min using a refrigerated Beckman Coulter Avanti J‐20‐XPI centrifuge equipped with a JLA 8.1 rotor and stored as described above.

#### 4.1.6 Protein Purifications

Protein purifications were performed at 4°C.

##### 4.1.6.1 H_6_‐RbsK

The pRbsK‐3 cell pellet was resuspended in Bind Buffer [10 mL, HEPES (50 mM, pH 7.5), containing NaCl (300 mM), and imidazole (10 mM)] with ~10 mg DNase I, lysozyme (1 mg/mL), and ~⅛ SIGMAFAST EDTA‐free protease inhibitor tablet. Cells were lysed by three sonication cycles using a Qsonica sonicator, and each cycle was 1 min, 2 s on, 2 s off, at 60% amplitude, and clarified by centrifugation at 40,000 x*g* and 4°C for 30 min. Cleared lysate was applied to a 1‐mL HisPur (Thermo Fisher) affinity column pre‐equilibrated with Bind Buffer, and fractions were collected by gravity. After all lysate was applied, the column was washed with 10 column volumes (CV, i.e., 10 mL) of Bind Buffer, 10 CV of Bind Buffer containing 8% Elution Buffer [HEPES (50 mM, pH 7.5), containing NaCl (300 mM), and imidazole (250 mM)]. Protein was eluted from the column by three fractions of 2 CV Elution Buffer. Elute fractions were analyzed by SDS‐PAGE (15% acrylamide gel) with Coomassie blue R staining, and fractions containing the target protein were pooled. Pooled protein was dialyzed twice against 1 L Storage Buffer (HEPES, 50 mM) containing NaCl (300 mM) at 4°C, flash frozen in liquid N_2_, and stored at −80°C.

##### 4.1.6.2 H_6_‐DeoK

The pDeoK‐3 cell pellet was suspended, lysed, and clarified as described for RbsK. The cleared lysates were applied to a 1‐mL HisTrap FF column (Cytiva) using an ÄKTA Pure FPLC System (Cytiva). The column was washed with 92% Bind Buffer and 8% Elution Buffer for 20 CV. Elution was performed with a 2 CV linear gradient to 100% Elution Buffer, then 6 CV of 100% Elution Buffer. The eluted product was collected in 2 mL fractions. The flow rate was 1.0 mL/min for all steps. Elution fractions were analyzed, pooled, dialyzed, frozen, and stored as described above.

##### 4.1.6.3 H_6_‐MBP‐RikA^WT^

The pRikA‐3 pellet was suspended in Bind Buffer (20 mL) containing DNase I (~25 mg), lysozyme (1 mg/mL), and ~¼ SIGMAFAST EDTA‐free protease inhibitor tablet. The suspension was lysed and the lysate clarified as described above. Cleared lysate was applied to a 1.5‐mL HisPur (Thermo Fisher) affinity column pre‐equilibrated with Bind Buffer, and fractions were collected by gravity. After all lysate was applied, the column was washed with 12 CV of Bind Buffer, 12 CV Wash Buffer 1 [HEPES (50 mM, pH 7.5) containing NaCl (300 mM), and imidazole (30 mM)], six CV Wash Buffer 2 [ HEPES (50 mM, pH 7.5), containing NaCl (300 mM), imidazole (50 mM)], and two CV Wash Buffer 3 [HEPES (50 mM, pH 7.5) containing NaCl (300 mM), imidazole (75 mM), and glycerol (10% v/v)]. Finally, H_6_‐MBP‐RikA was eluted in three fractions of one CV with H_6_‐MBP‐RikA Elution Buffer [HEPES (50 mM, pH 7.5) containing NaCl (300 mM), imidazole (250 mM), and glycerol (10% v/v)]. Elution fractions were analyzed and pooled as described above. Pooled protein was dialyzed twice against 1 L RikA Storage Buffer [HEPES (50 mM, pH 7.5) containing NaCl (300 mM), and glycerol (10% v/v)], then frozen and stored as described above.

##### 4.1.6.4 Variant H_6_‐MBP‐RikA^D343A^

The pRikA‐7 cell pellet was suspended, lysed, and clarified as described for RikA. The cleared lysates were applied to a 1‐mL HisPur (Thermo Fisher) affinity column pre‐equilibrated with Bind Buffer, and fractions were collected by gravity. After all lysate was applied, the column was washed with 18 CV of Bind Buffer, 18 CV Wash Buffer 1, 18 CV Wash Buffer 2, and four CV Wash Buffer 3. Finally, the H_6_‐MBP‐RikA^D343A^ protein was eluted with eight CV RikA Elution Buffer. This elute fraction was applied to a 0.5‐mL amylose resin column (NEB), which was washed 10 CV Amylose Wash Buffer [HEPES (50 mM, pH 7.5), containing NaCl (300 mM), glycerol (10% v/v)]. The final H_6_‐MBP‐RikA^D343A^ protein was eluted in two fractions of two CV Amylose Elute Buffer [HEPES (50 mM, pH 7.5) containing NaCl (300 mM), maltose (10 mM), and glycerol (10% v/v)]. Elution fractions were analyzed by SDS‐PAGE as described above, and fraction one was dialyzed and stored as described for H_6_‐MBP‐RikA^WT^.

##### 4.1.6.5 Protein Purity and Concentration Analyses

Protein purity was assessed as follows: 1:2 serial dilutions of each protein were prepared down to 1:8, separated by SDS‐PAGE (15% w/v acrylamide), and the gels visualized by Coomassie blue R staining. Purity percentage was calculated by densitometry using ImageJ (Schneider et al. 2012). Bands from these gels were excised and the identity of each was determined by trypsin digest and mass spectrometry performed by the Proteomics and Mass Spectrometry core facility at the University of Georgia. Protein concentrations were determined using the Qubit Protein Assay Kit (ThermoFisher) and a Qubit 4 fluorimeter following the manufacturer's protocol.

### 4.2 Kinase Assays

#### 4.2.1 Saturation Curves and Kinetic Analysis for Ribose and ATP

Kinetic parameters of each purified enzyme were determined using a coupled assay employing pyruvate kinase and lactate dehydrogenase (Kornberg and Pricer Jr. 1951). Reactions were on a clear 96‐well microplate (Falcon) with a final volume of 100 μL. Each reaction contained the following: HEPES (50 mM, pH 7.5), MgCl_2_ (10 mM), KCl (10 mM), NADH (3 mM), phosphoenolpyruvate (3 mM), pyruvate kinase (4.5 enzyme units; U = 1 μmol substrate consumed per minute), and lactate dehydrogenase (7 U). All enzymes were tested with each ribose and ATP in excess. For the reactions with excess ribose, concentration was 10 mM, and ATP concentrations are in Figure 6. For excess ATP, the H_6_‐MBP‐RikA reactions contained 3 mM ATP, while the H_6_‐RbsK and H_6_‐DeoK reactions had 250 μM ATP. Ribose concentrations are in Figure 6. The final concentrations of each protein in the reactions are as follows: 234 nM H_6_‐MBP‐RikA, 9.3 nM H_6_‐RbsK, or 391 nM H_6_‐DeoK. Reactions proceeded at 37°C with shaking, and the decrease in A_340 nm_ was monitored using a Spectramax Plus (Molecular Devices) or Eon (BioTek) microplate reader. All reactions were performed in technical triplicate two independent times. The rate of NADH consumption in each reaction was calculated in μM/min and plotted as a function of either ribose or ATP concentration. Michelis‐Menten and Hill Equation analyses were performed using Prism v10 (Graphpad) built‐in functions.

#### 4.2.2 Pentose Test

The ability of RikA to phosphorylate pentoses other than ribose was tested with the same method and conditions as described above with the following modifications. Each reaction contained ATP (2 mM), protein (234 nM), and 10 mM of the pentose tested. The pentoses tested were L‐arabinose, D‐deoxyribose, D‐xylose, and D‐xylulose. Each potential reaction was tested with H_6_‐MBP‐RikA^WT^ and H_6_‐MBP‐RikA^D343A^ proteins. The real concentration of full length H_6_‐MBP‐RikA^D343A^ was calculated based on the purity analysis such that 234 nM was the final concentration of the full‐length protein. BSA was added to the reactions containing H_6_‐MBP‐RikA^WT^ to a final concentration of 0.111 mg/mL to simulate the contamination in the H_6_‐MBP‐RikA^D343A^ preparation. Reactions were allowed to proceed for 3 h, and the rate was calculated using Prism v10 (Graphpad).

#### 4.2.3 *rikA* Expression From the Native Ribosome Binding Site (RBS)

The *rikA* gene and 278 base pairs upstream of the coding sequence were PCR amplified from strain JE6583 genomic DNA with Phusion High‐Fidelity DNA polymerase and primers RDM290 and RDM291. The product was verified with agarose gel electrophoresis then cleaned as described previously. This PCR product was amplified with primers RDM290 and RDM292, verified, and cleaned as described. The final PCR product contained 278 base pairs upstream of *rikA*, the *rikA* coding sequence with the codons for a triple glycine–alanine ((GA)_3_) spacer and hexahistidine (H_6_) tag at the 3′ end, and BspQI‐compatible overhangs on both ends. This product was cloned into plasmid pCV2 using BspQI and verified as described elsewhere (VanDrisse and Escalante‐Semerena 2016), yielding plasmid pRikA9, which was then transformed into strain JE28318 by electroporation.

The resulting strain was streaked to isolation on LB agar with ampicillin (100 μg/mL) and grown overnight at 37°C. A single colony was inoculated into nutrient broth (NB, Difco) with 100 μg/mL ampicillin and grown 18–20 h at 37°C with shaking at 180 rpm. The overnight culture was sub‐cultured 1:100 into 25 mL of no‐carbon essential (NCE) medium (Berkowitz et al. 1968) supplemented with D‐ribose (22 mM), MgSO_4_ (1 mM), Wolfe's trace minerals (Balch and Wolfe 1976), cyanocobalamin (15 nM), ampicillin (100 μg/mL), and into 25 mL of the same medium with L‐(+)‐arabinose (500 μM) added. Both cultures were grown for 18 h and harvested by centrifugation (10,000 × *g*, 10 min, 4°C).

Each pellet was resuspended in 1 mL Bind Buffer [HEPES (50 mM, pH 7.5), containing NaCl (300 mM), and imidazole (20 mM)] with ~⅛ SIGMAFAST EDTA‐free protease inhibitor tablet. Cells were lysed by three sonication cycles using a Qsonica sonicator, and each cycle was 1 min, 2 s on 2 s off, at 40% amplitude, and clarified by centrifugation at 16,100 × *g* and 4°C for 40 min. RikA‐(GA)_3_‐H_6_ was purified from each clarified cell lysate using NEBExpress Ni Spin Columns following the manufacturer's protocol. The wash buffer used contained HEPES (50 mM, pH 7.5), NaCl (300 mM), and imidazole (50 mM). The elution buffer contained HEPES (50 mM, pH 7.5), NaCl (300 mM), and imidazole (250 mM). The elution fractions for both the (+) arabinose and (−) arabinose conditions were analyzed by SDS‐PAGE as described previously. Bands from this gel were excised and the identity of each was determined by trypsin digest and mass spectrometry performed by the Proteomics and Mass Spectrometry core facility at the University of Georgia.

#### 4.2.4 Phylogenetic Analysis

We searched for RikA on the *fast.genomics* website (https://fast.genomics.lbl.gov) (Price and Arkin 2024) using its UniProt entry (Q8ZLG0, locus tag STM3547). The “gene neighborhoods” option was selected and the top 25 hits were displayed with the following options: 6Kb around the gene of interest displayed, show tree on, compact taxonomy, and color set to “≥ 2”. An SVG file of the resulting image was downloaded, and the label text was edited for legibility using Adobe Illustrator 2024. No other alterations were made.

## Author Contributions

**Aatif A. Jabbar:** investigation. **Regan D. McCormick:** conceptualization, validation, formal analysis, investigation, writing – original draft, writing – review and editing, visualization, supervision. **Jorge C. Escalante‐Semerena:** conceptualization, validation, formal analysis, supervision, resources, funding acquisition, visualization, project administration, writing – review and editing.

## Funding

This work was supported by the National Institute of General Medical Sciences (R35GM130399).

## Ethics Statement

The authors have nothing to report.

## Conflicts of Interest

The authors declare no conflicts of interest.

## Supporting information


**Figure S1:** Ribose kinase single deletions grow to full density on ribose as a sole carbon source. Each strain was grown overnight in NB + ampicillin (100 μg/mL) at 37°C with agitation and sub‐cultured 1:100 into NCE minimal medium with either (A) ribose (22 mM), or (B) deoxyribose (22 mM) as the sole carbon source. Cultures were grown in 96‐well plates at 37°C with agitation. Genetic notations are as follows: *rbsK::kan* ^+^ is *rbsK83::kan* ^+^, *deoK::kan* ^+^ is *deoK407::kan* ^+^. ‘Vector’ stands for the empty cloning vector pCV1 that contains an arabinose‐inducible promoter. Plasmids were maintained with ampicillin (100 μg/mL), and ectopic gene expression was induced with L‐(+)‐arabinose (500 μM). This experiment was conducted in technical triplicate of biological duplicates. Error bars represent one standard deviation from the mean. Error bars that are not visible are smaller than the symbol. Deletions of either *rbsK*, *deoK*, or *rikA* do not prevent growth of *S*. Typhimurium on ribose. Deletion of *deoK*, however, abolishes growth using deoxyribose as the sole carbon source.
**Figure S2:** Ribose kinase deletions show no defects on glucose as a sole carbon source. Each strain was grown overnight in NB + ampicillin (100 μg/mL) at 37°C with agitation and sub‐cultured 1:100 into NCE minimal medium with glucose (18 mM) as the sole carbon source. Cultures were grown in 96‐well plates at 37°C with agitation. Genetic notations are as follows: *rbsK::kan* ^+^ is *rbsK83::kan* ^+^, *deoK::kan* ^+^ is *deoK407::kan* ^+^, Δ*rbsK* is Δ*rbsK84*, Δ*deoK* is Δ*deoK408*, ΔRK3 is the *ΔrbsK84* Δ*deoK408 rikA::cat* ^+^ triple deletion. Each strain carries the empty cloning vector pCV1 that contains an arabinose‐inducible promoter. Plasmids were maintained with ampicillin (100 μg/mL), and ectopic gene expression was induced with L‐(+)‐arabinose (500 μM). This experiment was conducted in technical triplicate of biological duplicates. Error bars represent one standard deviation from the mean. Error bars that are not visible are smaller than the symbol. Ribose kinase deletions in any combination do not affect growth on glucose as the sole carbon source.
**Figure S3:** Purity gels of H_6_‐RbsK, H_6_‐DeoK, H_6_‐MBP‐RikA, and H_6_‐MBP‐RikA_D343A_. The proteins were purified as described in *Experimental procedures*. Serial 1:2 dilutions of each protein were analyzed by SDS‐PAGE with Coomassie blue R staining. The total amount of protein in picomoles is indicated above each lane, black arrows point towards the target protein, and the bands outlined with red rectangles were identified as degradation products of the target protein by trypsin digestion and mass spectrometry. Precision Plus protein molecular weight ladder (BioRad) was used for each gel. Percentage purity was calculated by densitometry in ImageJ, and the percent purity of each full‐length protein is as follows: 80.8% H_6_‐RbsK, 92.5% H_6_‐DeoK, 86.9% H_6_‐MBPRikA, and 13.7% H_6_‐MBP‐RikA D343A.
**Figure S4:** The H_6_‐MBP tag does not interfere with RikA activity in vivo. Each strain was grown overnight in NB + ampicillin (100 μg/mL) at 37°C with agitation and subcultured 1:100 into NCE minimal medium with ribose (22 mM) as the sole carbon source. Cultures were grown in 96‐well plates at 37°C with agitation. ΔRK3 is the *ΔrbsK84* Δ*deoK408 rikA::cat* ^+^ triple deletion. “Vector” stands for the empty cloning vector pCV1 that contains an arabinose‐inducible promoter, pRikA^WT^ is pRikA‐2, pH_6_‐MBP‐RikA is pRikA‐4 (See *Experimental procedures*). Plasmids were maintained with ampicillin (100 μg/mL), and ectopic gene expression was induced with L‐(+)‐arabinose (500 μM). This experiment was conducted in technical triplicate of biological duplicates. Error bars represent one standard deviation from the mean. Error bars that are not visible are smaller than the symbol. H_6_‐MBP‐RikA has ribose kinase activity in vivo.
**Figure S5:** Protein recovered in Figure 7A are identified as RikA‐(GA)_3_‐H_6_. Mass spectra and peak lists of protein excised from the gel in Figure 7A following trypsin digestion and mass spectrometry (MALDI‐TOF) analysis. (A) RikA protein primary sequence showing the putative initiating methionine (M1) and the initiating methionine found in this study (M90). Colors identify peptides identified by the mass spectrum shown below. The colors in panel A match those in panel B. The C terminal hexahistidine tag is identified by the green bar. A linker of alternating Gly and Ala residues was designed to ensure availability of the H_6_ tag to the nickel resin used in the purification (see *Experimental procedures*). (B) Protein recovered from JE28887 cells grown on ribose minimal medium without arabinose. The peaks from each protein band were analyzed using MS‐FIT (https://prospector.ucsf.edu/prospector/cgi‐bin/msform.cgi?form=msfitstandard) with the following settings: Database “User Protein”, User Protein Sequence was the full predicted sequence of RikA‐(GA)_3_‐H_6_, “Tol” set to 0.6 Da, no Constant Mods, “Oxidation of M” in Possible Modifications. Both samples were identified as RikA.

## Data Availability

All data generated during this work are reported in this paper and its Supporting Information file.
