# Supplementary material for: Protein STM3547 From Salmonella typhimurium Is a Phosphofructose Kinase B‐Type Enzyme With Ribose Kinase Activity
Source: Mol Microbiol. 2026 Jun 5;126(2):129–44. doi: 10.1111/mmi.70084 (PMC13432705; doi:10.1111/mmi.70084)
Supplement: Supplementary file 1 — Figure S1: Ribose kinase single deletions grow to full density on ribose as a sole carbon source. Each strain was grown overnight in NB + ampicillin (100 μg/mL) at 37°C with agitation and sub‐cultured 1:100 into NCE minimal medium with either (A) ribose (22 mM), or (B) deoxyribose (22 mM) as the sole carbon source. Cultures were grown in 96‐well plates at 37°C with agitation. Genetic notations are as follows: rbsK::kan + is rbsK83::kan +, deoK::kan + is deoK407::kan +. ‘Vector’ stands for the empty cloning vector pCV1 that contains an arabinose‐inducible promoter. Plasmids were maintained with ampicillin (100 μg/mL), and ectopic gene expression was induced with L‐(+)‐arabinose (500 μM). This experiment was conducted in technical triplicate of biological duplicates. Error bars represent one standard deviation from the mean. Error bars that are not visible are smaller than the symbol. Deletions of either rbsK, deoK, or rikA do not prevent growth of S. Typhimurium on ribose. Deletion of deoK, however, abolishes growth using deoxyribose as the sole carbon source. Figure S2: Ribose kinase deletions show no defects on glucose as a sole carbon source. Each strain was grown overnight in NB + ampicillin (100 μg/mL) at 37°C with agitation and sub‐cultured 1:100 into NCE minimal medium with glucose (18 mM) as the sole carbon source. Cultures were grown in 96‐well plates at 37°C with agitation. Genetic notations are as follows: rbsK::kan + is rbsK83::kan +, deoK::kan + is deoK407::kan +, ΔrbsK is ΔrbsK84, ΔdeoK is ΔdeoK408, ΔRK3 is the ΔrbsK84 ΔdeoK408 rikA::cat + triple deletion. Each strain carries the empty cloning vector pCV1 that contains an arabinose‐inducible promoter. Plasmids were maintained with ampicillin (100 μg/mL), and ectopic gene expression was induced with L‐(+)‐arabinose (500 μM). This experiment was conducted in technical triplicate of biological duplicates. Error bars represent one standard deviation from the mean. Error bars that are not visible are smaller [file MMI-126-129-s001.pdf]

Supporting Information

Protein STM3547 from *Salmonella* Typhimurium is a phosphofructose kinase B-type enzyme with ribose kinase activity

Regan D. McCormick[1], Aatif A. Jabbar[1], and Jorge C. Escalante-Semerena[1*]
[1]Department of Microbiology, University of Georgia, Athens USA

*Corresponding author: 330G Cedar Street Building C, 136 Cedar Street, Athens, GA 30602 USA; Email: jcescala@uga.edu

Running title: studies of a new ribose kinase in *Salmonella* Typhimurium

Keywords: carbohydrate metabolism, *Salmonella enterica*, ribose kinase, PfkB family of sugar kinases

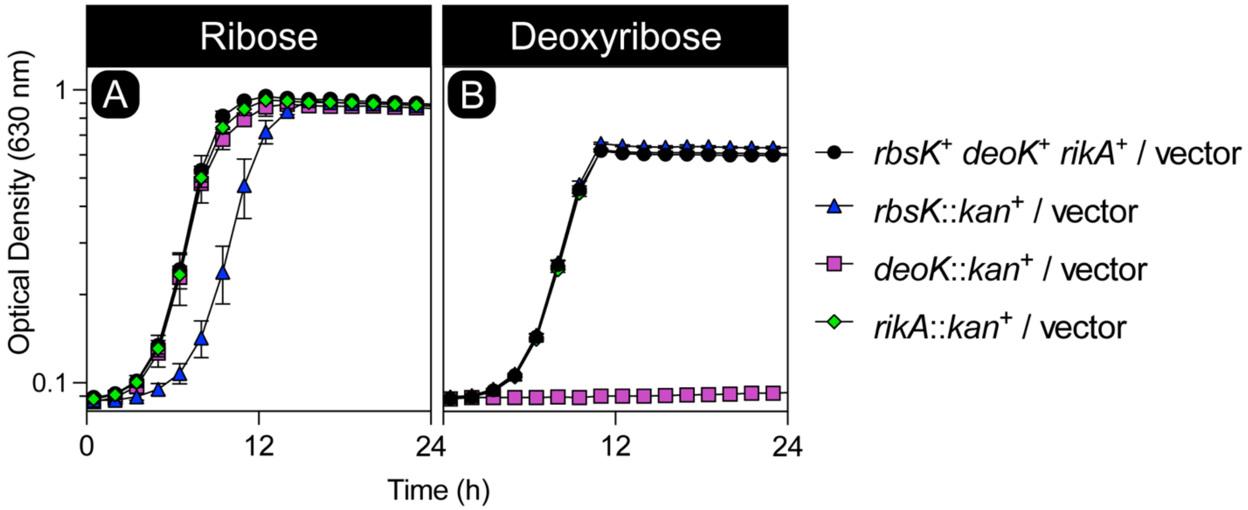

Figure S1. Ribose kinase single deletions grow to full density on ribose as a sole carbon source. Each strain was grown overnight in NB + ampicillin (100 µg/mL) at 37°C with agitation and sub-cultured 1:100 into NCE minimal medium with either **(A)** ribose (22 mM), or **(B)** deoxyribose (22 mM) as the sole carbon source. Cultures were grown in 96-well plates at 37°C with agitation. Genetic notations are as follows: *rbsK*::*kan*$^+$ is *rbsK83*::*kan*$^+$, *deoK*::*kan*$^+$ is *deoK407*::*kan*$^+$. 'Vector' stands for the empty cloning vector pCV1 that contains an arabinose-inducible promoter. Plasmids were maintained with ampicillin (100 µg/mL), and ectopic gene expression was induced with L-(+)-arabinose (500 µM). This experiment was conducted in technical triplicate of biological duplicates. Error bars represent one standard deviation from the mean. Error bars that are not visible are smaller than the symbol. Deletions of either *rbsK*, *deoK*, or *rikA* do not prevent growth of *S.* Typhimurium on ribose. Deletion of *deoK*, however, abolishes growth using deoxyribose as the sole carbon source.

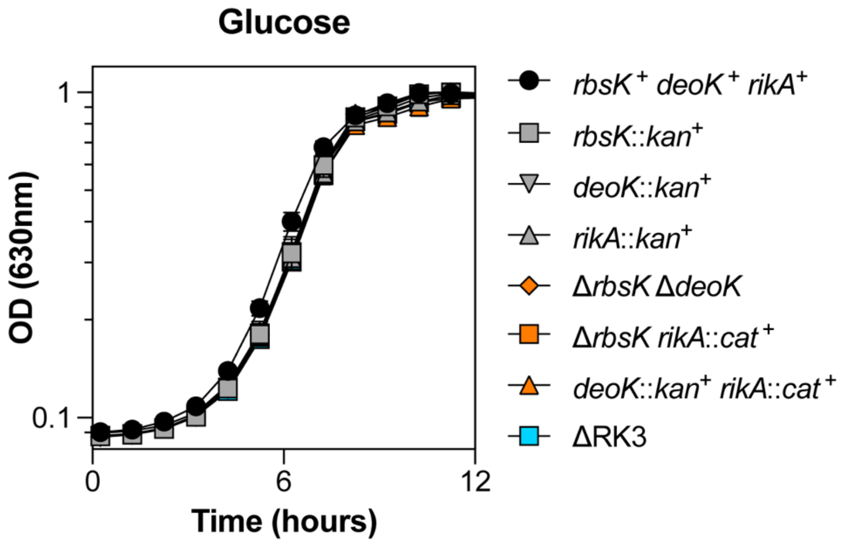

Figure S2. Ribose kinase deletions show no defects on glucose as a sole carbon source. Each strain was grown overnight in NB + ampicillin (100 µg/mL) at 37°C with agitation and sub-cultured 1:100 into NCE minimal medium with glucose (18 mM) as the sole carbon source. Cultures were grown in 96-well plates at 37°C with agitation. Genetic notations are as follows: *rbsK*::*kan*$^+$ is *rbsK83*::*kan*$^+$, *deoK*::*kan*$^+$ is *deoK407*::*kan*$^+$, Δ*rbsK* is Δ*rbsK84*, Δ*deoK* is Δ*deoK408*, ΔRK3 is the Δ*rbsK84* Δ*deoK408* *rikA*::*cat*$^+$ triple deletion. Each strain carries the empty cloning vector pCV1 that contains an arabinose-inducible promoter. Plasmids were maintained with ampicillin (100 µg/mL), and ectopic gene expression was induced with L-(+)-arabinose (500 µM). This experiment was conducted in technical triplicate of biological duplicates. Error bars represent one standard deviation from the mean. Error bars that are not visible are smaller than the symbol. Ribose kinase deletions in any combination do not affect growth on glucose as the sole carbon source.

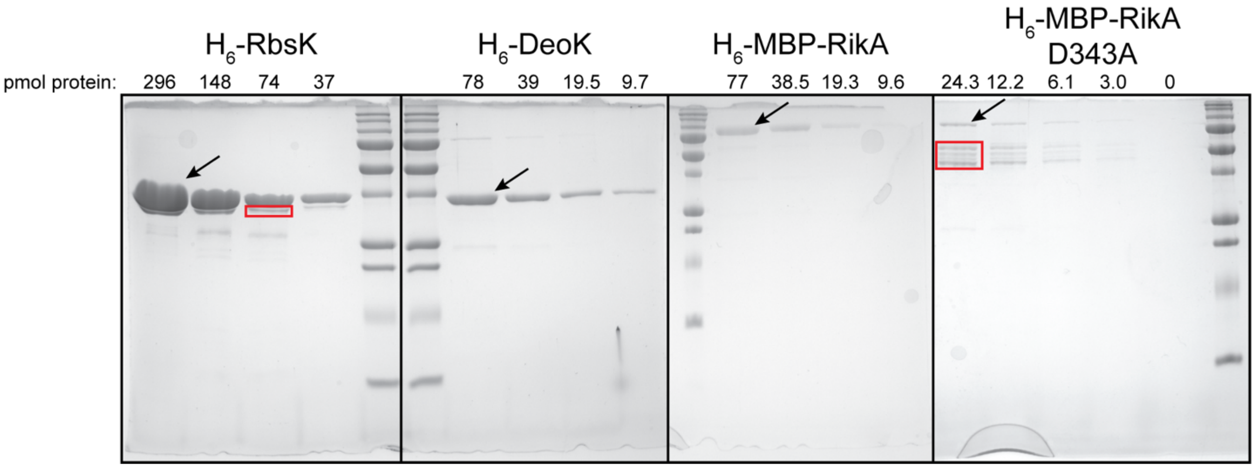

Figure S3. Purity gels of H_6-RbsK, H_6-DeoK, H_6-MBP-RikA, and H_6-MBP-RikA^{D343A}. The proteins were purified as described in *Experimental Procedures*. Serial 1:2 dilutions of each protein were analyzed by SDS-PAGE with Coomassie blue R staining. The total amount of protein in picomoles is indicated above each lane, black arrows point towards the target protein, and the bands outlined with red rectangles were identified as degradation products of the target protein by trypsin digestion and mass spectrometry. Precision Plus protein molecular weight ladder (BioRad) was used for each gel. Percentage purity was calculated by densitometry in ImageJ, and the percent purity of each full-length protein is as follows: 80.8% H_6-RbsK, 92.5% H_6-DeoK, 86.9% H_6-MBP-RikA, and 13.7% H_6-MBP-RikA D343A.

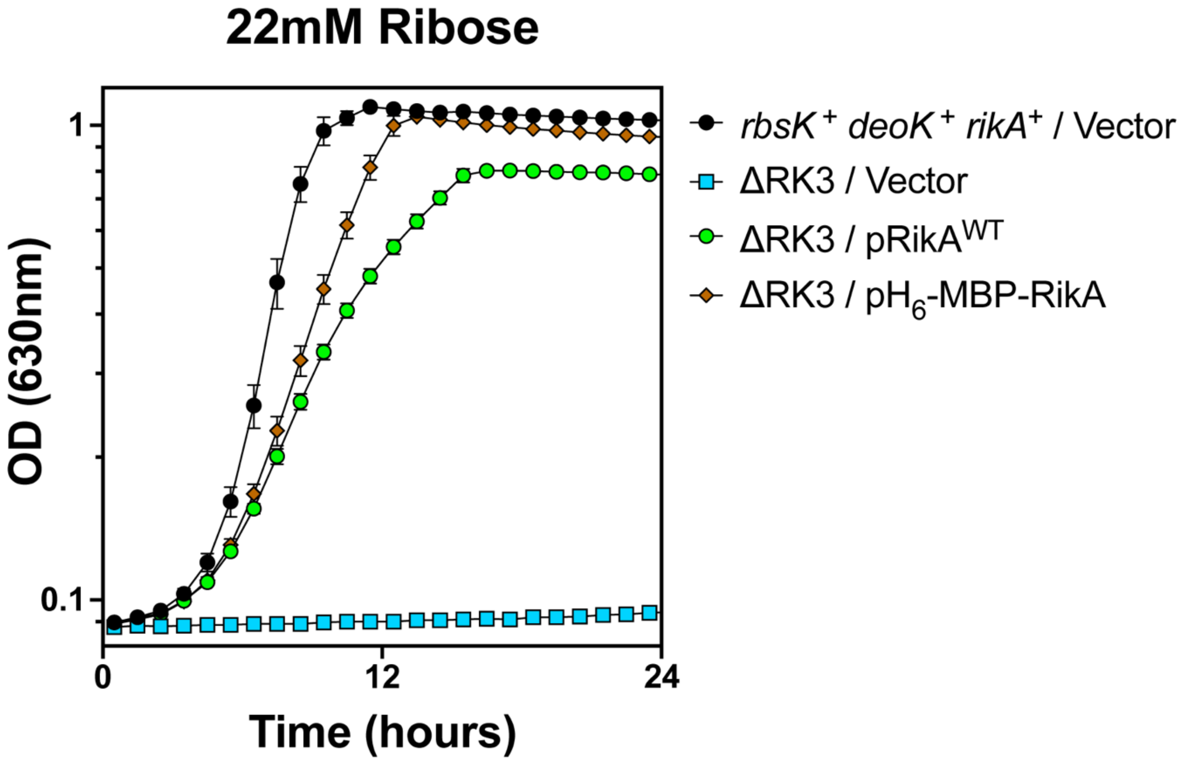

Figure S4. The H_6-MBP tag does not interfere with RikA activity *in vivo*. Each strain was grown overnight in NB + ampicillin (100 µg/mL) at 37°C with agitation and subcultured 1:100 into NCE minimal medium with ribose (22 mM) as the sole carbon source. Cultures were grown in 96-well plates at 37°C with agitation. ΔRK3 is the Δ*rbsK84* Δ*deoK408* *rikA*::*cat*$^+$ triple deletion. “Vector” stands for the empty cloning vector pCV1 that contains an arabinose-inducible promoter, pRikA^{WT} is pRikA-2, pH_6-MBP-RikA is pRikA-4 (See *Experimental Procedures*). Plasmids were maintained with ampicillin (100 µg/mL), and ectopic gene expression was induced with L-(+)-arabinose (500 µM). This experiment was conducted in technical triplicate of biological duplicates. Error bars represent one standard deviation from the mean. Error bars that are not visible are smaller than the symbol. H_6-MBP-RikA has ribose kinase activity *in vivo*.

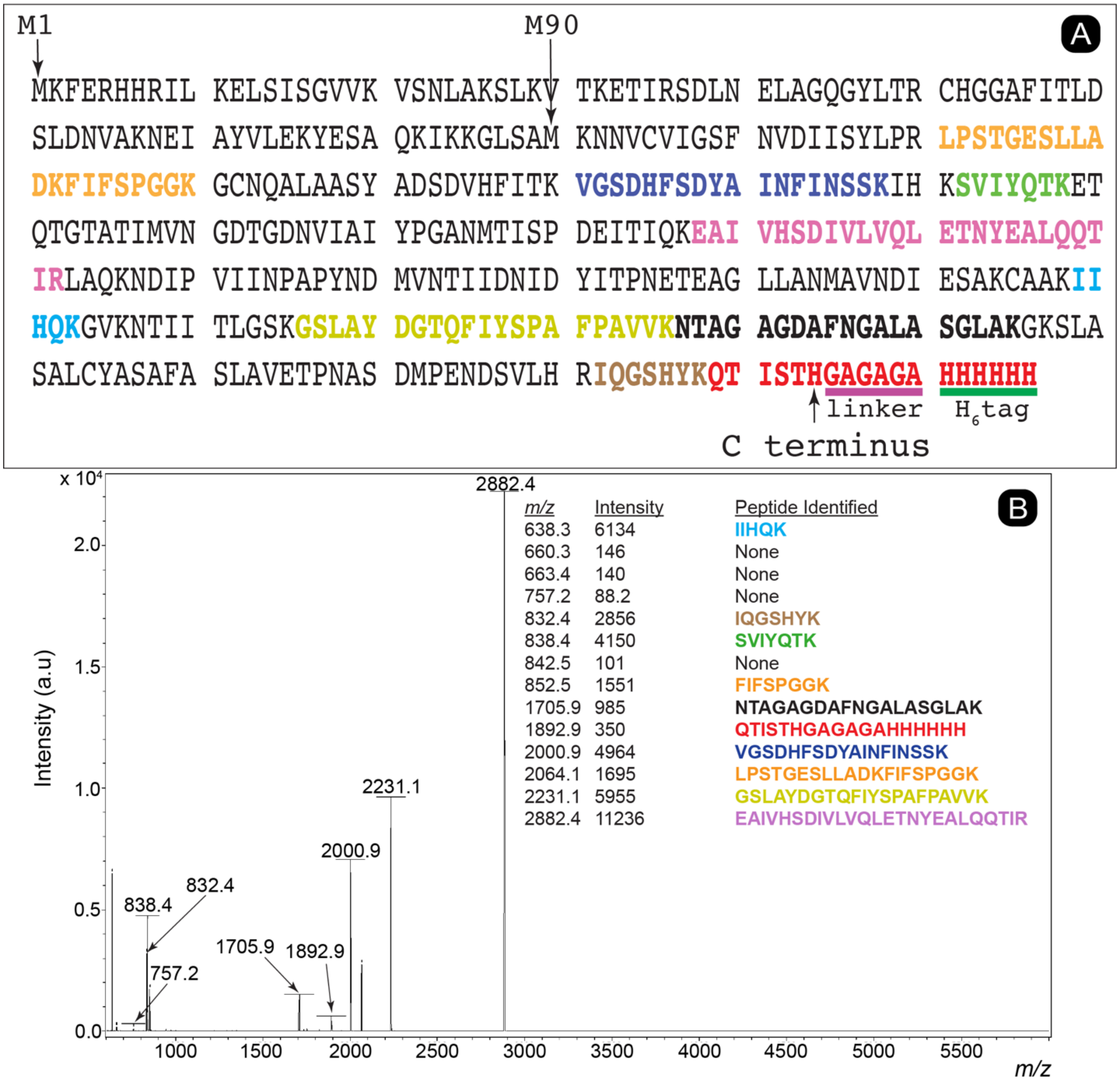

Figure S5. Protein recovered in Figure 7A are identified as RikA-(GA)$_3$-H$_6$. Mass spectra and peak lists of protein excised from the gel in Figure 7A following trypsin digestion and mass spectrometry (MALDI-TOF) analysis. **(A)** RikA protein primary sequence showing the putative initiating methionine (M1) and the initiating methionine found in this study (M90). Colors identify peptides identified by the mass spectrum shown below. The colors in panel A match those in panel B. The C terminal hexahistidine tag is identified by the green bar. A linker of alternating Gly and Ala residues was designed to ensure availability of the H$_6$ tag to the nickel resin used in the purification (see *Experimental procedures*). **(B)** Protein recovered from JE28887 cells grown on ribose minimal medium without arabinose. The peaks from each protein band were analyzed using MS-FIT (https://prospector.ucsf.edu/prospector/cgi-bin/msform.cgi?form=msfitstandard) with the following settings: Database “User Protein”, User Protein Sequence was the full predicted sequence of RikA-(GA)$_3$-H$_6$, “Tol” set to 0.6 Da, no Constant Mods, “Oxidation of M” in Possible Modifications. Both samples were identified as RikA.
